# Cabazitaxel-induced ureteritis in metastatic castration-resistant prostate cancer patients: a single center case series 2014–2024

**DOI:** 10.1186/s40644-025-00872-2

**Published:** 2025-04-09

**Authors:** Ahmed H. Zedan, Jesper S. Gade, Karsten Egbert Arnold Zieger, Mads H. Poulsen, Anja Schmidt Vejlgaard, Filip Lund Hjorth Fredensborg

**Affiliations:** 1 Department of Oncology, Lillebaelt Hospital, Vejle Hospital, University Hospital of Southern Denmark, Vejle, Denmark; 2 Department of Radiology, Lillebaelt Hospital, Vejle Hospital, University Hospital of Southern Denmark, Vejle, Denmark; 3 Department of Urology, Lillebaelt Hospital, Vejle Hospital, University Hospital of Southern Denmark, Vejle, Denmark; 4 Department of Urology, Esbjerg and Grindsted Hospital, University Hospital of Southern Denmark, Esbjerg, Denmark; 5 Department of Regional Health Research, University of Southern Denmark, Odense, Denmark

**Keywords:** Cabazitaxel, Ureteritis, Hematuria, Hydronephrosis, mCRPC, Case series

## Abstract

**Background:**

One of the main and effective therapy choices for patients with metastatic castration-resistant prostate cancer (mCRPC) is cabazitaxel (CBZ). Cystitis and hematuria are among the most significant non-hematological adverse events associated with CBZ treatment. But because the prevalence of CBZ-induced ureteritis has not been thoroughly studied, this case series investigation was carried out to emphasize the condition’s clinical relevance and potential treatment alternatives.

**Case presentation:**

Between June 2014 and May 2024, 354 patients diagnosed with mCRPC were treated with CBZ at the Department of Oncology, Vejle Hospital. A total of 36 patients (10%) exhibited ureteritis-like symptoms, presenting with discomfort in the pelvis, lower abdomen, or flanks, with or without hematuria. Radiological evidence of ureter changes was present in 29 out of 36 individuals (80%), along with hydronephrosis/hydroureter in some patients. Prior to therapy with CBZ, radiation to the pelvis or lower abdomen was documented in 7 out of 36 patients (19%). Various analgesics and dosage modifications were considered for the therapy of CBZ-induced ureteritis, with treatment discontinuation yielding the most favorable results.

**Conclusion:**

The onset of ureteritis during CBZ treatment is an underrated side effect in clinical practice. Hematuria and hydronephrosis/hydroureter are the most associated complications. Both analgesics and dosage reduction should be contemplated for management, while therapy cessation may be requisite in certain individuals.

**Supplementary Information:**

The online version contains supplementary material available at 10.1186/s40644-025-00872-2.

## Background

Cabazitaxel (CBZ) in combination with prednisone has been a standard treatment option for patients with metastatic castration-resistant prostate cancer (mCRPC) in the post-docetaxel setting since its endorsement by the Food and Drug Administration and European Medical Agency in 2010–2011 [1, 2].

As a taxane, CBZ primarily inhibits the microtubular network’s mitotic and interphase cellular functions in order to exert its antineoplastic effects [3]. While CBZ treatment is generally well tolerated by mCRPC patients, drug-related adverse events (AEs) such as inflammation of the urinary tract, including cystitis, and hematuria have been reported [4]. These AEs have the potential to cause treatment interruptions or even discontinuation [5].

The ureters are bilateral muscular tubular structures with a diameter of about 3 mm, serving to connect the kidneys to the urinary bladder and transport urine from the renal pelvis to the bladder [6]. The histology of the ureter is characterized by three different layers: the mucosa, composed of transitional epithelium and supported by submucosal connective tissue, known as the lamina propria, muscular layer and outer adventitia. These three layers of the normal ureter are indistinguishable on CT scans. Nonetheless, noninfectious inflammation often leads to thickening of the ureteral wall, especially affecting the periureteral tissues, and the enhancement of the ureter wall with contrast is observed when an intravenous agent is administered [7].

Drug-induced ureteritis has been previously documented in relation to antibiotics [8], immunotherapy [9, 10], and chemotherapy [11], including CBZ [12, 13]. The precise mechanism of CBZ-induced ureteritis is not fully understood, as CBZ is primarily metabolized in the liver (> 95%) by CYP3A (80–90%). Seven plasma metabolites (including 3 active O-demethylation metabolites) were found, with the major one accounting for 5% of parent exposure. Approximately 20 metabolites of CBZ are eliminated in urine and feces. Following a 1-hour intravenous infusion of CBZ at a dosage of 25 mg/m2 in patients, around 80% of the administered dose was eliminated within a 2-week period. CBZ is eliminated mainly through feces as various metabolites, accounting for 76% of the administered dose [14].

Some observations have indicated that prior treatment with radiation therapy (RT) in the pelvic region could potentially increase the risk of CBZ-induced inflammation in the urinary tract system [15], known as radiation recall syndrome [16]. Nevertheless, other case-report studies have reported cases of CBZ-induced ureteritis in patients who have not undergone pelvic radiation [12]. Despite being a well-known phenomenon to oncologists since its initial description approximately 70 years ago [17], radiation recall syndrome remains poorly understood. Its histological and clinical characteristics often exhibit acute or chronic inflammatory reactions localized to previously irradiated regions, induced by the introduction of precipitating systemic drugs after RT [18]. These occurrences can manifest in various tissues, such as the skin [19], lungs [20], gastrointestinal system [21], genitourinary tract [22], and even the central nervous system [18]. Taxanes, anthracyclines, and antimetabolites are among the chemotherapies for which radiation recall syndrome have been most frequently reported [23, 24, 25]. Radiation recall syndrome can occur months or years after irradiation, suggesting that the mechanisms involved may differ from those associated with radiosensitization [26], even though some researchers argue that radiation recall syndrome is a form of delayed radiosensitization [27].

The incidence and clinical significance of CBZ-induced ureteritis in real-world settings have not been adequately investigated. Therefore, we conducted a case series study to illustrate the impact of CBZ-induced ureteritis and provide practical recommendations for its management in everyday clinical practice.

## Patients and methods

Patients diagnosed with mCRPC who underwent treatment with at least one cycle of CBZ (regardless treatment-line) at the Department of Oncology, Vejle Hospital, during the period of June 2014 to May 2024 were retrospectively identified using a combination of both mCRPC code (DC619Z), and CBZ code (BWHA263) in the National Patient Register (Landspatientregisteret (LPR)).

The study was approved by The Danish Data Protection Agency according to Danish law (24/39851).

The electronic medical records (EMRs) of all patients were reviewed retrospectively by AHZ, FLHF, and ASV to collect the clinical data of medical history (including any previous treatment with RT in the pelvis), treatment history. The patient’s EMR was thoroughly examined to document any event of pain in the pelvis, lower abdomen, or flanks during the treatment period with CBZ in order to establish a correlation to the urinary tract.

The radiological findings of any abnormalities in the urinary tract were investigated in relation to the patients’ symptoms of urinary tract inflammation. All scans from those who experienced symptoms mimicking ureteritis during CBZ treatment were retrospectively reassessed by an independent uroradiologist (JSG), with particular attention to any alterations in the ureters, while blinded from their original assessment. Routine scans closest to the onset of symptoms were reassessed, and if signs of ureteritis were present, the following scan was reassessed as a follow-up.

Furthermore, we collected information regarding the management of ureteritis, such as medical treatment with analgesics, surgical intervention (with or without biopsy from the urinary tract), and disruption/discontinuation of CBZ.

## Results

From June 2014 to May 2024, 354 patients were treated with CBZ (25 mg/m^2^) at our institution. A cut-off date for data collection was 29. Oct. 2024. Median CBZ cycles given per patient were 6 cycles (1–41). All patients in this cohort were treated by Docetaxel prior to CBZ, either in mCRPC setting, or recently in metastatic castration sensitive prostate cancer alone or in combination with Abiraterone. All patients were evaluated biochemically before every treatment cycle (every 3 weeks), and radiologically every 3–4 cycles; for bone metastases by bone scintigraphy (replaced by 18 F Sodium Fluoride positron emission tomography (NaF/PET) scan since 2019) as well as chest and abdomen computed tomography (CT) for soft tissue metastases.

In this study, ureteritis was defined radiologically as an evident diffuse circumferential thickening of the ureteral wall, accompanied by contrast enhancement of the wall (with or without periureteral fat stranding) observed on intravenous contrast-enhanced CT scans.

A total of 36 patients (10%) reported discomfort in the pelvis/lower abdomen or flanks following the initiation of CBZ treatment. From EMR data, radiographic findings of ureter abnormalities were present in 16 out of 36 patients (44%). Furthermore, 13 patients (36%) exhibited signs of ureter abnormalities upon blind retrospective reassessment of their routine scans at the onset of symptoms.

The suspicion of ureteritis was supported by contrast-enhanced CT imaging in the portal venous phase. The most common observation was mild wall thickening of the ureter, associated with visual contrast enhancement, along with varying degrees of stranding in the periureteral fat.

In 22 out of 36 patients (60%), radiological signs of varying grades of hydronephrosis or hydronephroureter were observed, of whom 20 patients (90%) exhibited radiological abnormalities in the ureter as well.

Surgical intervention with either a double-J catheter or nephrostomy catheter, was deemed necessary in 4 out of 22 patients exhibiting radiological evidence of hydronephrosis/hydroureter. Additionally, one patient was managed with a double-J catheter just to address symptoms of ureteritis. The primary indications for surgical intervention were the progression of hydronephrosis or the deterioration of renal function. After surgery all five patients demonstrated improvement in either renal function or hydronephrosis, along with a reduction in flank pain symptoms.

The overall incidence of hematuria, both microscopic and macroscopic, in the whole group was 18%. Among the 36 patients exhibiting pelvic or flank discomfort, 19 (53%) presented with hematuria. Radiological evidence of ureteral abnormalities was seen in almost all cases (18 individuals).

Of all 36 patients, only 24 exhibited signs or symptoms of a urinary tract infection. However, only one patient had a positive urine culture.

About 19% of patients (7 out of 36) with pelvic pain had undergone either curative intent or palliative radiation to the pelvis or lower abdomen before receiving CBZ treatment. A biopsy from the urinary bladder was performed in 7 out of 36 patients, with signs of inflammation observed in three patients (43%). No patients had a biopsy performed from the ureter.

Among the 36 patients who reported pain or discomfort in the pelvic region, lower abdomen, or flanks, treatment with CBZ remained consistent for 26 patients, of whom five (19%) reported pain improvement solely through analgesics. In four patients, dose reduction was implemented, resulting in pain relief for only one patient (25%), who also received analgesics. Treatment was entirely discontinued in six patients, resulting in the complete resolution of pelvic pain symptoms, regardless of analgesic use.

The analgesics predominantly prescribed by the patient’s medical oncologist included paracetamol (77%), non-steroidal anti-inflammatory drugs (NSAIDs) (50%), and opioids (36%), utilized either as monotherapy or in combination.

Follow-up scans indicated that radiological changes in the ureter resolved completely or partially in 17 out of 26 patients (60%) within a median time of 2.8 months (range, 0.7–14.2).

**Fig. 1:**
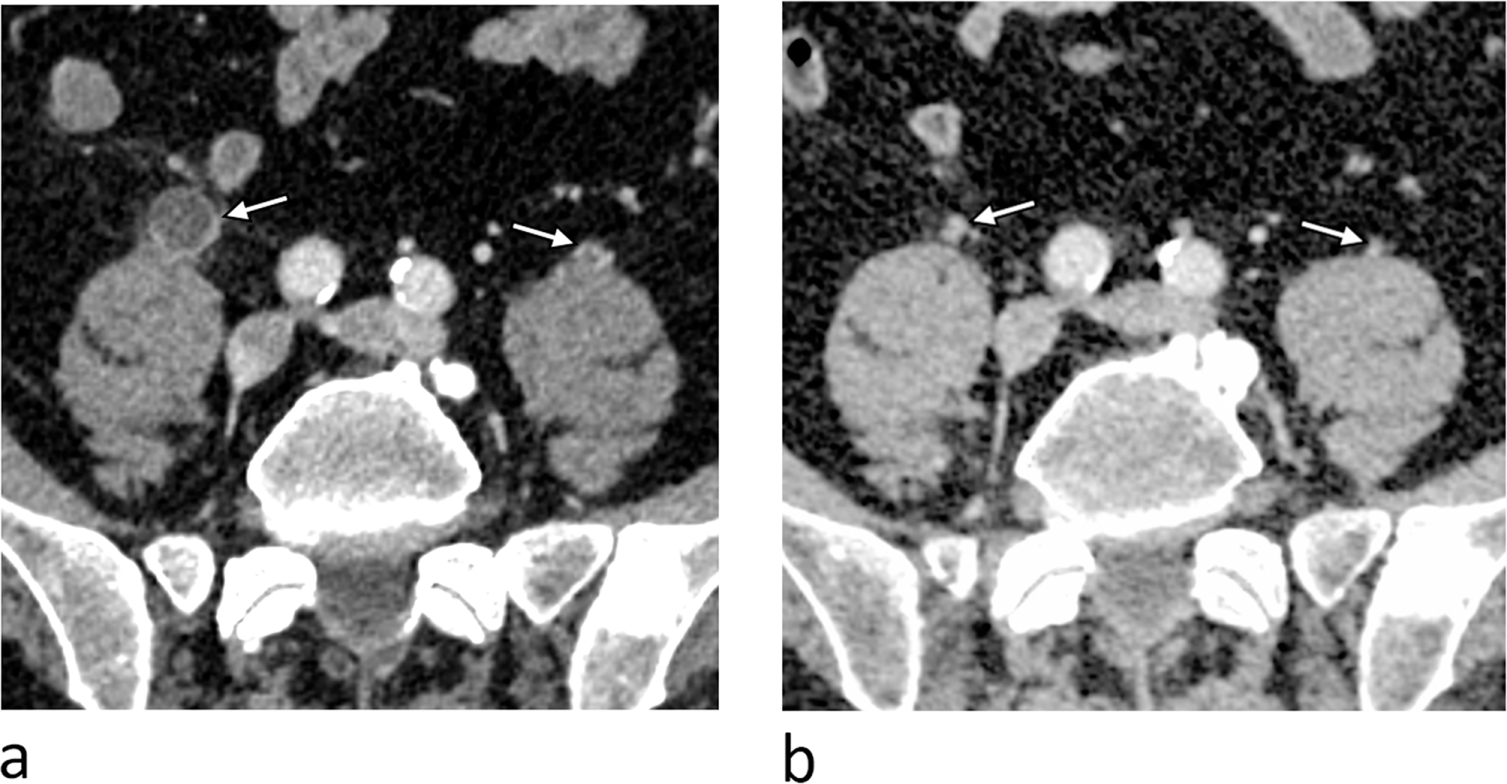
Abdominal CT with contrast in early porto venous phase, axial images. (**a**) At the onset of symptoms there is visible contrast enhancement in mildly thickened walls of both ureters (arrows), the right ureter is dilated and there is some stranding of the periureteral fat. These are radiologic findings consistens with ureteritis. (**b**) At the follow-up CT, there is complete remission of wall thickening and perifokal stranding of the ureters (arrows)

**Fig. 2:**
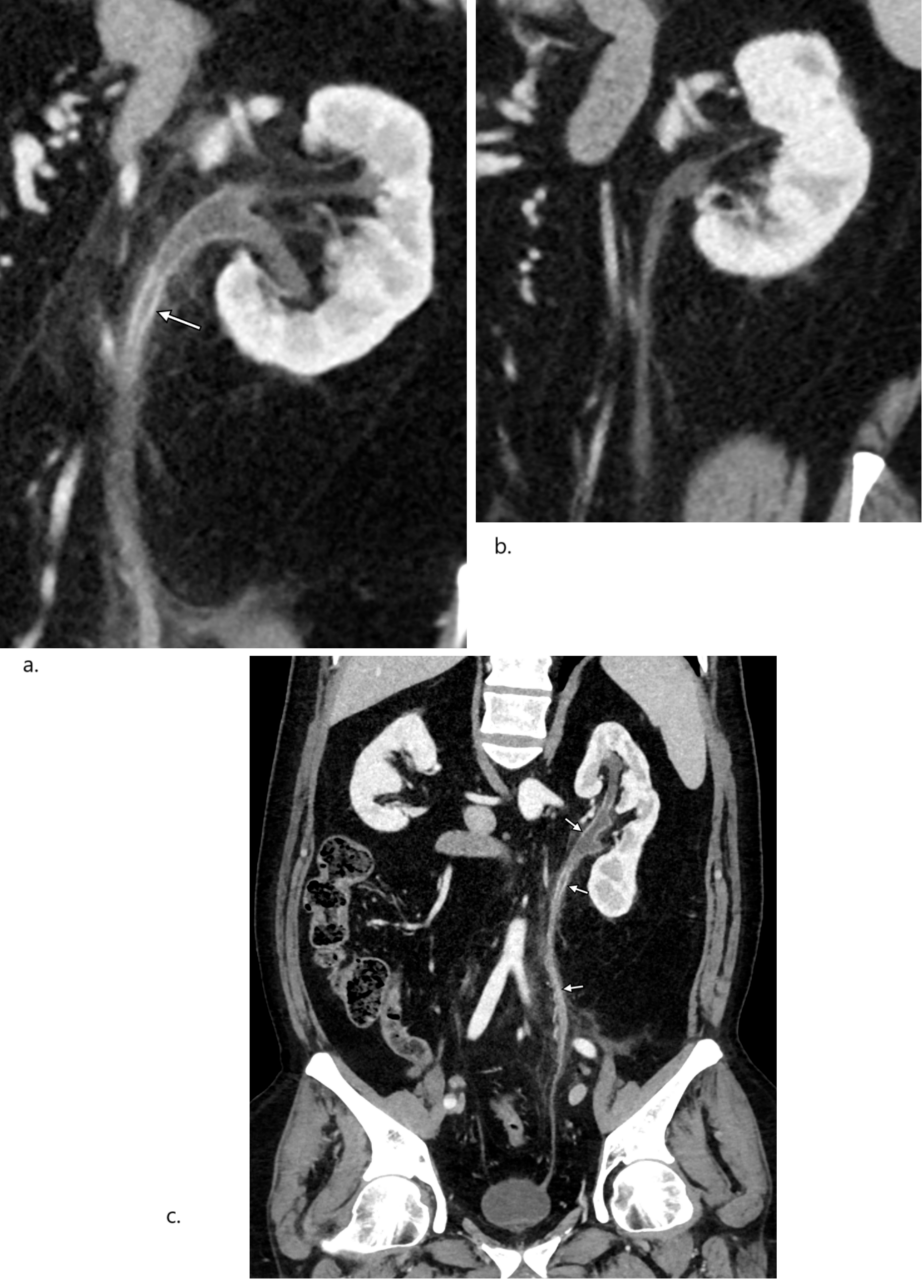
Abdominal CT with contrast in early porto venous phase, paracoronal images. (**a**) Mild to moderate wall thickening with contrast enhancement in extensive areas of left ureter (arrow) and pelvicalyceal system, consistent with ureteritis. (**b**) Follow-up scan shows total or near to total remission of inflammatory changes in the left ureter. (**c**) Curved reconstruction, from CT at the time of onset of symptoms showing the extension of the inflammatory changes in left ureter and pelvicalyceal system (arrows) – most of the cranial two thirds of the system is affected

Figures 1 and 2 illustrate the alterations in the ureter corresponding to the onset of symptoms and subsequent follow-up scans for two patients.

The complete clinical and radiological data for the 36 patients are presented in Table S1.

## Discussion

In this case series study, approximately 10% of patients showed symptoms similar to ureteritis during CBZ treatment at our facility. At the onset of symptoms, 80% had some degree of radiological changes in the ureter consistent with ureteritis. Hydronephrosis and hematuria were the most common accompanying signs and symptoms to ureteritis in 60% and 53% of patients, respectively. The use of analgesics alone (paracetamol, NSAIDs, or opioids) was successful in managing pain in 20% of the cases. However, discontinuing treatment with CBZ was shown to be the most effective approach, as it resulted in complete relief of symptoms and signs of ureteritis in 100% of the patients.

While no cases of CBZ-induced ureteritis have been documented, several urinary tract-related AEs that potentially mirror ureteritis symptoms have been observed in many clinical trials investigating CBZ in the mCRPC population [4, 5, 28, 29]. Hematuria and abdominal pain were among the most common non-hematological AEs in the TROPIC study, accounting for 17% and 12%, respectively [4]. While abdominal pain was not reported in the PROSELICA trial [28], the FIRSTANA study, showed a rate of 8–10% [5]. A similar incidence of abdominal pain (8%) was recorded in a CARD trial in participants treated with a CBZ dosage of 25 mg/m^2^ [29].

The development of ureteritis during CBZ treatment in this case series study does not seem to be dose-dependent. This is in accordance with the FIRSTANA study, which demonstrated that the incidence of abdominal pain was higher in patients treated with the 20 mg/m2 arm than in those which received a higher dose of 25 mg/m2, at 10% vs. 8%, respectively, irrespective to the number of cycles given to patients in each arm [5].

Drug-induced ureteritis has been documented in a number of case reports involving cancer patients. Two cases of gastric cancer treated with an immunotherapy-based regimen (sintilimab in the first case and nivolumab in the second case) demonstrated clinical and radiological signs of ureteritis, which resolved completely following a brief course of corticosteroid therapy [10]. Activated cytotoxic T-cells resulting from immunotherapy may target normal urothelium, suggesting a potential mechanism for immunotherapy-induced ureteritis [30].

Cyclophosphamide-based combination chemotherapy has been described to cause symptoms and signs of ureteritis in a case of ovarian cancer [11]. The primary pathway for the elimination of cyclophosphamide is via the kidneys, leading to urological toxicity as a common side effect, notably hemorrhagic cystitis [31].

In patients with mCRPC, two prior case studies [13, 32] and one case series [15] have reported the incidence of ureteritis caused by CBZ. The metabolism of CBZ mostly occurs in the liver, with most of the given dosage eliminated via feces, whereas renal excretion of CBZ and its metabolites accounts for less than 4% of the total dose [14], so making the fundamental mechanism of CBZ-induced ureteritis unclear. A hypothesis suggests that prior radiation to the pelvic region may elucidate the mechanism of ureteritis known as radiation recall syndrome [15, 32]. In our study, less than 20% of patients exhibiting radiological evidence of ureteritis had undergone radiation therapy at varying doses to the pelvis and lower abdomen prior to CBZ treatment. Kosaka et al. also reported a case of ureteritis due to CBZ in a mCRPC patient with no prior history of radiation treatment [12]. This suggests that CBZ may be the key factor causing irritation in the ureteral mucosa.

Hydronephrosis, with or without hydroureter, was the most prevalent complication associated with CBZ-induced ureteritis in our cohort. Over 60% of patients have reported improvement or complete regression of hydronephrosis within a median duration of 2.8 months, either spontaneously or following urological intervention.

The prevalence of hematuria has varied between 15% and 25% in the PROSELICA, FIRSTANA, and CARD trials. Furthermore, in the French registry study FUJI (Follow-Up of Jevtana in Real Life), the incidence of hematuria of all grades was similarly 20% [33]. This aligns with our data, indicating an incidence of hematuria of around 24%. In our study, hematuria was one of the most prevalent symptoms occurring concurrently or within a few weeks after the beginning of pelvic or flank discomfort. In over 50% of patients who had urinary bladder biopsy for hematuria management, evidence of inflammation in the mucosal wall were seen, suggesting similar alterations may have occurred in the ureteral mucosa, however not confirmed histologically.

Approximately 28% of patients exhibiting definite radiographic evidence of CBZ-induced ureteritis have undergone either dosage adjustment or complete cessation of CBZ therapy in our study. This aligns with both the CARD and FIRSTANA trials, which reported that CBZ medication was discontinued owing to adverse events in 20% and 25–32% of patients, respectively [5, 29].

The optimal management of CBZ-induced ureteritis involves treatment withdrawal, although this may be an undesirable option in certain clinical contexts. Consequently, dose modification combined with various analgesics under careful supervision may serve as a viable alternative strategy. Although Geethal et al. [15] did not report a recurrence of symptoms upon re-initiation of CBZ in two cases, we conducted a rechallenge of CBZ for one patient in our center, which resulted in an immediate recurrence of both clinical and radiological signs of CBZ-induced ureteritis.

The administration of sulfated glycosaminoglycans, such as chondroitin sulfate, and non-sulfated glycosaminoglycans, such as hyaluronic acid, has shown potential in the treatment and prevention of urinary tract infections [34]. Hyaluronic acid, similar to chondroitin sulfate, is essential for preserving tissue integrity and provide structural support to the lining of the urinary system [35]. Additionally, hyaluronic acid has anti-inflammatory characteristics that may regulate the inflammatory response induced by an infection, therefore minimizing tissue damage and accelerating recovery [36]. Therefore, these two agents could be investigated as potential remedies in the management of CBZ-induced ureteritis.

In some patients in this study, CT urography, which involves a CT scan with contrast-filled ureters, was conducted to enhance visualization of the ureters. The mild thickening and contrast enhancement of the ureters indicative of ureteritis were obscured by blooming effects from the highly dense contrast within the lumen. Therefore, CT urography is inadequate for assessing ureteritis in this clinical setting. A CT scan with contrast in the venous or potentially late arterial phase is most appropriate for this purpose.

This study represents the largest case series examining ureteritis induced by CBZ. Furthermore, this study has contributed to the improvement of management for CBZ-induced ureteritis at our center, with an increasing focus on the presenting symptoms as well as both the urological and radiological findings related to this critical clinical challenge. Nonetheless, several pitfalls have encountered our study. The primary limitations of this study are the retrospective data collection and the nonspecific pain history recorded in EMRs. The majority of mCRPC patients in this study presented with bone metastases, including those in the pelvic and vertebral regions, which may resemble pain caused by ureteritis. The absence of systematic follow-up for patients with CBZ-induced ureteritis has hindered a comprehensive understanding and management of this significant clinical challenge associated with CBZ treatment.

## Conclusion

This study emphasizes the clinical importance of CBZ-induced ureteritis in patients diagnosed with mCRPC as a common side effect during the treatment period by CBZ. Despite renal/urinary pain being one of the most frequently reported AEs associated with CBZ treatment, it has not received significant attention in daily clinical practice. An improved understanding and diagnosis of this critical adverse event could assist clinicians in managing and guiding patients whose oncological treatment options are already constrained at this stage.

## Electronic supplementary material

Below is the link to the electronic supplementary material.

Supplementary Material 1

Supplementary Material 2

## Data Availability

No datasets were generated or analysed during the current study.

## Abbreviations

AEs: Adverse events
CBZ: Cabazitaxel
CT: Computed tomography
EMRs: Electronic medical records
FUJI: Follow-up of jevtana in real life
LPR: Landspatientregisteret
mCRPC: Metastatic castration-resistant prostate cancer
NaF/PET: 18 F sodium fluoride positron emission tomography
NSAIDs: Non-steroidal anti-inflammatory drugs
RT: Radiation therapy
